# Digital Cell Atlas of Mouse Uterus: From Regenerative Stage to Maturational Stage

**DOI:** 10.3389/fgene.2022.847646

**Published:** 2022-05-20

**Authors:** Leyi Zhang, Wenying Long, Wanwan Xu, Xiuying Chen, Xiaofeng Zhao, Bingbing Wu

**Affiliations:** ^1^ The Fourth Affiliated Hospital, Zhejiang University School of Medicine, Yiwu, China; ^2^ Key Laboratory of Tumor Microenvironment and Immune Therapy of Zhejiang Province, Second Affiliated Hospital, Zhejiang University School of Medicine, Hangzhou, China; ^3^ Cancer Institute (Key Laboratory of Cancer Prevention & Intervention, National Ministry of Education), Second Affiliated Hospital, Zhejiang University School of Medicine, Hangzhou, China; ^4^ Department of Breast Surgery, Second Affiliated Hospital, Zhejiang University School of Medicine, Hangzhou, China

**Keywords:** endometrium, single-cell RNA sequencing, estrus cycle, mononuclear phagocyte system, endometrial mesenchymal stem cells

## Abstract

Endometrium undergoes repeated repair and regeneration during the menstrual cycle. Previous attempts using gene expression data to define the menstrual cycle failed to come to an agreement. Here we used single-cell RNA sequencing data of C57BL/6J mice uteri to construct a novel integrated cell atlas of mice uteri from the regenerative endometrium to the maturational endometrium at the single-cell level, providing a more accurate cytological-based elucidation for the changes that occurred in the endometrium during the estrus cycle. Based on the expression levels of proliferating cell nuclear antigen, differentially expressed genes, and gene ontology terms, we delineated in detail the transitions of epithelial cells, stromal cells, and immune cells that happened during the estrus cycle. The transcription factors that shaped the differentiation of the mononuclear phagocyte system had been proposed, being *Mafb*, *Irf7*, and *Nr4a1*. The amounts and functions of immune cells varied sharply in two stages, especially NK cells and macrophages. We also found putative uterus tissue-resident macrophages and identified potential endometrial mesenchymal stem cells (high expression of *Cd34*, *Pdgfrb*, *Aldh1a2*) *in vivo*. The cell atlas of mice uteri presented here would improve our understanding of the transitions that occurred in the endometrium from the regenerative endometrium to the maturational endometrium. With the assistance of a normal cell atlas as a reference, we may identify morphologically unaffected abnormalities in future clinical practice. Cautions would be needed when adopting our conclusions, for the limited number of mice that participated in this study may affect the strength of our conclusions.

## Introduction

Proper implantation of the embryo in the maternal endometrium is critical for a normal pregnancy, and the sophisticated transformation of the endometrium in the three different menstrual states is regulated by the collaboration of cell populations under the influence of hormones (Kumar et al., 2011).

The attempt to use gene expression to define the menstrual cycle has been seen in previous studies, which reported a strong relationship existing between histopathology and transcriptional profiles of the samples and validated the importance of using molecular profiles to evaluate the endometrial status (Haber et al., 2017). Other than humans, the endometria of mice, rats, and cows have also been analyzed to a certain extent (Reese et al., 2001; Naciff et al., 2002). However, there was little consistency between these microarray-based studies, for the differentially expressed genes reported in each study showed large variability. These molecular profiles remained at the tissue level. The fact that the proportions of the epithelium, stroma, immune cells, and blood vessels in individual specimens were different may cause the variability. However, single-cell analysis can solve this obstacle, as it allows us to detect cell-to-cell variability, possible subpopulations, and rare cell types.

The estrous cycle in mice averages 4–5 days and is a repetitive but dynamic process, reflecting changes in the levels of estradiol and progesterone secreted by the ovarian follicles (Cora et al., 2015). There are different criteria for defining the estrus cycle of mice (Vidal and Filgo, 2017), but no matter how the cycle is defined, mice uteri undergo the same hormone change patterns and the recurrences of regeneration and maturation as humans. In order to match with the recurring physiologic changes in humans, we divided the estrous cycle of mice into two stages: the regeneration stage and the maturation stage. The regenerative stage amounted to the proliferative phase in humans and proestrus and estrus phases in mice, the maturational stage amounted to the secretory phase in humans and the metestrus and the diestrus phases in mice. In doing so, we could seek insights and inspirations from mice single-cell RNA sequencing (scRNA-seq) data to shed light on human endometrium research.

The cognition of the immune conditions in the menstrual cycle has been updated rapidly, yet the amounts or the functional traits of multiple immune cell types are still under debate. The immune cells in decidua have been analyzed thoroughly (Jiang et al., 2018). In sharp contrast, the features of immune cells in the regenerative stage and the maturational stage have not gained enough attention. Up to today, the complicated hormone-induced immune regulation has left a huge riddle for us to solve (Kumar et al., 2014).

Based on the research gaps mentioned above, we aimed to construct a cell atlas of mice uteri including multiple cell types at the single-cell level. We used published scRNA-seq data of two C57BL/6J mice uteri to build an integrated cell atlas from the regenerative endometrium to the maturational endometrium, and elucidated the transitions that happened in epithelial cells, stromal cells, and immune cells during the estrous cycle, hoping to provide new insights into cell dynamics in the uterus and provide a normal reference for future studies under pathologic conditions.

## Materials and Methods

### Datasets Selection and Data Processing

Two datasets from public databases were enrolled in this study. GSE108097 from Gene Expression Omnibus (https://www.ncbi.nlm.nih.gov/geo/, RRID: SCR_005012), containing scRNA-seq data of 3,756 cells from two 6-to-10-week-old female C57BL/6J mice uteri, was selected to build the digital mouse cell atlas of mouse uterus (Han et al., 2018). Mouse 1 contained 2041 cells, and mouse 2 contained 1715 cells. Based on the expression levels of proliferating cell nuclear antigen, differentially expressed genes of two samples, and gene ontology terms, we concluded that mouse 1 was in the maturational stage and mouse 2 was in the regeneration stage. Another scRNA-seq data of endometrial tissue was obtained from a previous study to compare the cultured endometrial stromal cells (human endometrial cells in the secretory stage were collected and cultured for 8 days in the cell culture medium consisting of a 1:1 mixture of DMEM (Cat No E15-892, GE Healthcare, United States)/Ham’s F12 (Cat No E15-890, GE Healthcare), supplemented with 10% FBS and Antibiotic-Antimycotic solution (GE Healthcare) and uncultured ones (endometrial biopsies collected from women in the secretory stage) (Krjutškov et al., 2016). We used R software (version 3.6.3; http://www.Rproject.org, RRID: SCR_001905) and Seurat package of R (version 2.3, https://satijalab.org/seurat/, RRID: SCR_016341) (Satija et al., 2015) to process the data. Seurat is a computational strategy to process and explore scRNA-seq data. The filter criteria of cells were determined as default. It is not necessary to obtain ethical approval because we used published online datasets.

### Computational Bioinformatics Analyses of Single-Cell RNA Sequencing Data

Principal component analysis (PCA), uniform manifold approximation and projection (UMAP), t-distributed stochastic neighbor embedding (tSNE), and visualization were performed by the Seurat package of R (Satija et al., 2015). Seurat is a computational strategy to process and explore scRNA-seq data. The fate decisions and pseudotime trajectories of endometrium stromal cells and the mononuclear phagocyte system were reconstructed by the Monocle package of R (version 2.10.1, http://cole-trapnell-lab.github.io/monocle-release/docs/, RRID: SCR_016339) (Cole et al., 2014; Qiu et al., 2017; Wu et al., 2017). In short, Monocle performs differential expression and time-series analysis for single-cell expression experiments, which orders individual cells according to progress through a biological process, without knowing ahead of time which genes define progress through that process. Possible stem cells identification was performed by RaceID3 (https://github.com/dgrun/RaceID, RRID: SCR_017045) and StemID2 (https://github.com/dgrun/StemID, RRID: SCR_017242) (Grün et al., 2015). RaceID3 is an algorithm for rare cell type identification in complex populations of single cells, while StemID2 is an algorithm based on RaceID3 for the inference of differentiation trajectories and the prediction of the stem cell identity.

### Functional Enrichment Analyses

Gene Ontology (GO) analyses were conducted through clusterProfiler (clusterProfiler, RRID: SCR_016884) (Yu et al., 2012; Walter et al., 2015) and online GO resource website (Ashburner et al., 2000; 2019). Statistically significant GO terms (*p* < 0.05) were identified.

### Histology and Immunofluorescence

Histology and immunofluorescence were conducted according to our previous procedures (Wu et al., 2019). Briefly, vagina smears were firstly used to determine the mouse estrous cycle (Supplementary Figure S1) (Bertolin and Murphy, 2014). Then mouse uterine tissues were collected and fixed in 4% (w/v) paraformaldehyde, and then dehydrated in an ethanol gradient. Then the paraffin sections of 10 μm thickness were stained with hematoxylin and eosin. Immunostaining was carried out as follows: The series of 10 μm-thick sections were rehydrated, fixed with 4% (w/v) paraformaldehyde for 30 min, antigen retrieval was conducted by incubating in citrate antigen retrieval solution at 65°C overnight, rinsed three times with PBS, and treated with blocking solution (1% BSA) for 30 min, prior to incubation with primary antibodies at 4°C overnight. The primary antibodies rabbit anti-mouse antibodies against KRT7 (Abcam, ab181598), the primary antibodies mouse anti-mouse antibodies against PCNA (Abcam, ab29), the primary antibodies rat anti-mouse antibodies against CD34 (Biolegend, 119307) were used to detect the expression of selected proteins within the uterine tissues. The goat anti-rat-cy3 secondary antibody (Beyotime Biotechnology, A0507), goat anti-rabbit-488 secondary antibody (Invitrogen, A11008), donkey anti-mouse 546 secondary antibody (Invitrogen, A10036), and DAPI (Beyotime Biotechnology, C1002) were used to visualize the respective primary antibodies and the cell nuclei. All procedures were carried out according to the manufacturer’s instructions.

### Statistical Analysis

All statistical analyses were performed by Graphpad Prism 8.0 software (https://www.graphpad.com/, RRID: SCR_002798) and R software (version 3.6.3; http://www.Rproject.org, RRID: SCR_001905). A two-sided probability value of *p* < 0.05 was considered being statistically significant.

## Results

### Single-Cell RNA Sequencing Analysis of Two Mice Uteri

**FIGURE 1:**
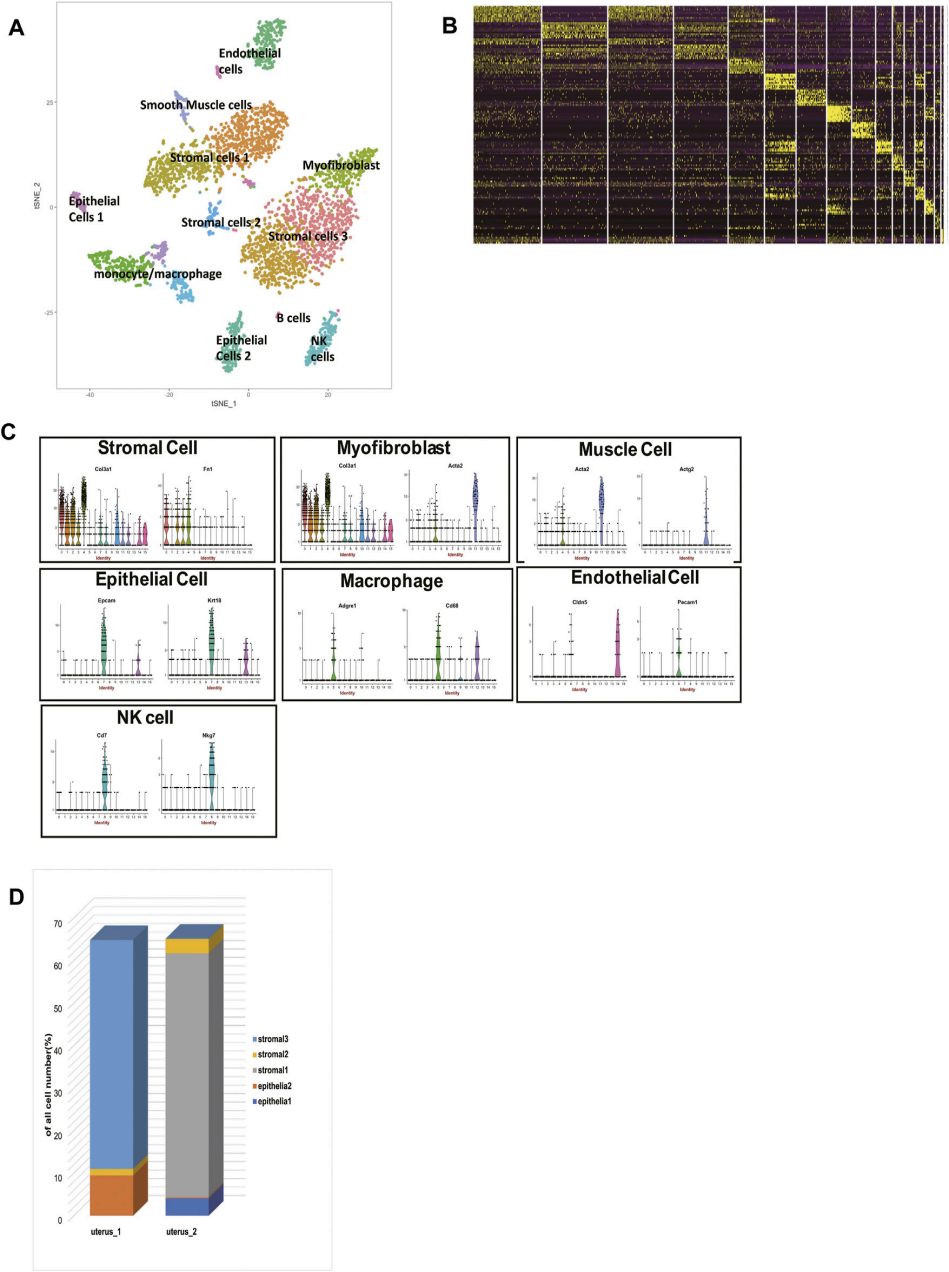
single-cell RNA sequencing analysis of two mice uteri. **(A)** t-distributed stochastic neighbor embedding (t-SNE) diagram of two mice uteri. **(B)** Heatmap of single cells from the two uteri revealed 16 populations. **(C)** Violin plots indicating the expression of marker genes of each cell cluster. **(D)** The cell composition of the two uteri was significantly different. T-SNE: t-distributed stochastic neighbor embedding.

The scRNA-seq data of two 6-to-10-week-old female C57BL/6J mice were processed using published Seurat pipelines (Satija et al., 2015; Han et al., 2018). In total, we analyzed 3,756 single cells and identified 16 cell clusters, which were grouped into eight major cell types (Figures 1A,B, Supplementary Table S1). Then we clarified the identities of each cluster (Figure 1C, Supplementary Table S2). Cluster 0, cluster 1, cluster 2, cluster 3, cluster 10 all highly expressed *Col3a1* and *Fn1*, so we clarified them into stromal cells. Cluster 4, in the meantime, specifically expressed *Acta2*, which made us clarify them into myofibroblasts. Cluster 11, however, expressed *Acta2* and *Actg2* without *Col3a1*, so cells in cluster 11 belonged to muscle cells. Cluster 5, cluster 9, and cluster 12 all highly expressed *Cd68* and *Adgre1*, so they were macrophages/monocytes. *Cd7* and *Nkg7* could be found highly expressed in cluster 8, which made cluster 8 natural killer (NK) cells. *Ly6d* and *Cd79a* were highly expressed in cluster 15, so we identified cluster 15 as B cells. Cluster 13 and cluster 7 both had high expression levels of *Krt8*, *Krt18,* and *Epcam,* making them epithelial cells. Cluster 6 and cluster 14 had high expression levels of *Cldn5* and *Pecam*, making them endothelial cells. In this way, we clarified the identities of 16 cell clusters, building a solid foundation for further analysis.

We identified 5 sub-clusters of stromal cells (cluster 0, cluster 1, cluster 2, cluster 3, cluster 10) and 2 sub-clusters of epithelial cells (cluster 13 and cluster 7). According to their distances in the t-SNE, we divided the 5 sub-clusters of stromal cells into three groups: stromal cells 1, stromal cells 2, and stromal cells 3. The proportions of each cell type in two mice were shown (Figure 1D). Surprisingly, we found that stromal cells 1 were exclusively in mouse 2, and stromal cells 3 were exclusively in mouse 1. Stromal cells 2 could be found both in mouse 1 and mouse 2. This phenomenon was also seen in epithelial cells. Epithelial cells 1 were exclusively in mouse 2, while epithelial cells 2 were exclusively in mouse 1.

### Comparison of Epithelial Cells and Stromal Cells Revealed Two Uteri of Distinct Estrus Stages

**FIGURE 2:**
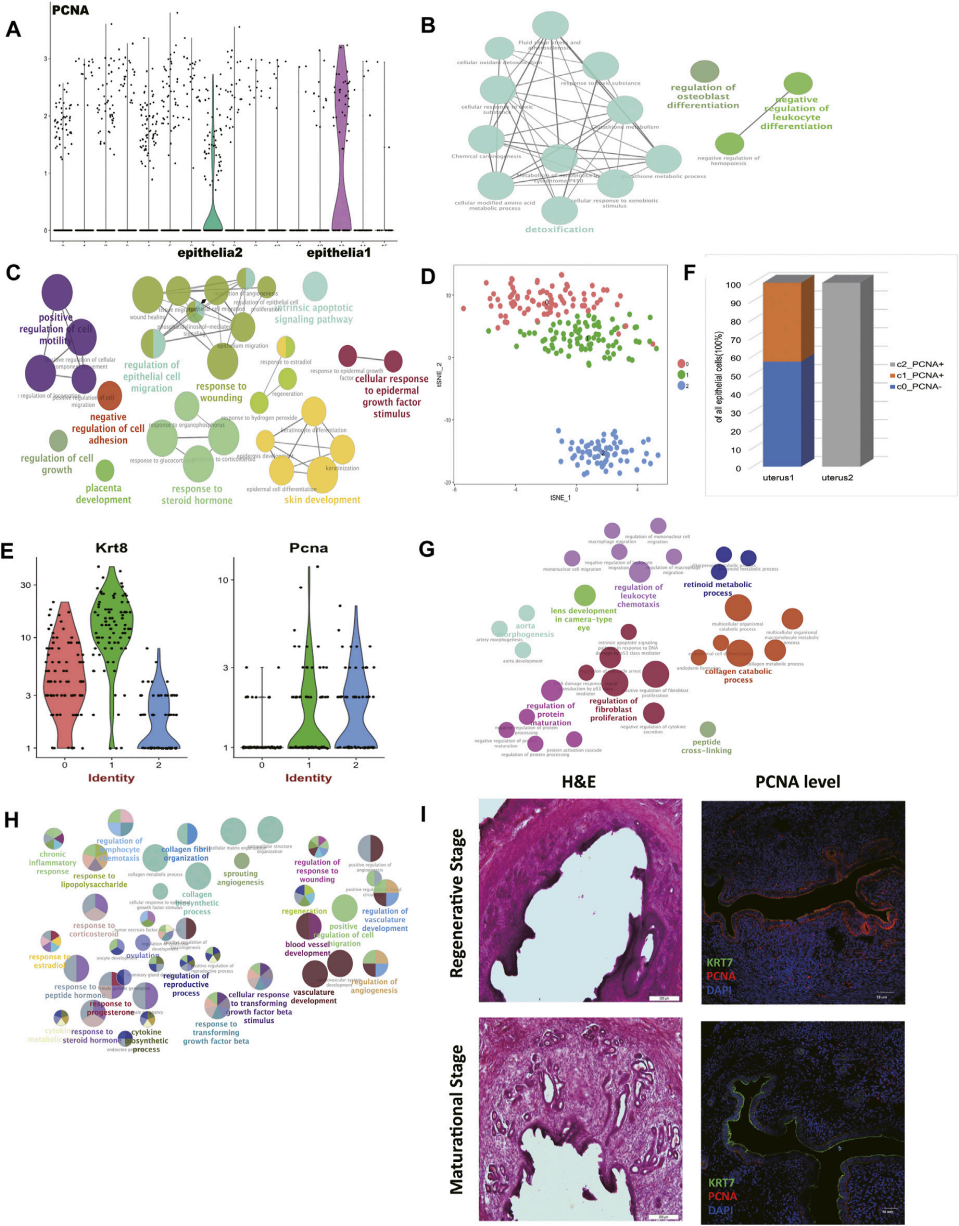
Comparison of epithelial cells and stromal cells revealed two uteri of distinct estrus stages. **(A)** *Pcna* was highly expressed in epithelial cells 1 compared to epithelial cells 2. Gene ontology (GO) analysis of differentially upregulated genes in **(B)** epithelial cells 1 and **(C)** epithelial cells 2. **(D)** t-distributed stochastic neighbor embedding (t-SNE) diagram of epithelial cells in two mice uteri. **(E)** *Pcna* was highly expressed in sub-cluster 1 and sub-cluster 2. **(F)** The epithelial cell composition of the two uteri was significantly different. Gene ontology (GO) analysis of differentially upregulated genes of stromal cells in **(G)** the regenerative stage and **(H)** the maturational stage. **(I)** Representative H&E and IF staining of PCNA expression levels in mice endometrium. (DAPI, blue; PCNA, red; KRT7, green). Scale bars, 200 μm (H&E) and 50 μm (IF). GO: Gene ontology; t-SNE: t-distributed stochastic neighbor embedding; PCNA: proliferating cell nuclear antigen; IF: immunofluorescence; H&E: hematoxylin and eosin.

We believed the different cell contents of two mice had further biological significance. We found that proliferating cell nuclear antigen (*Pcna*) was highly expressed in epithelial cells 1 (mouse 2) (Figure 2A). The protein encoded by this gene is a cofactor of DNA polymerase delta, which is regarded as the marker of proliferation (Celis and Celis, 1985). Previous studies have reported that for humans experiencing estrous cycles, the divergence of the expression level of *Pcna* was mainly due to the states of the estrous cycle rather than age. It has been reported that the expression of *Pcna* showed a high peak in the proliferative phase, and then decreased sharply in the secretory phase (Li et al., 1993; Noci et al., 1995; Hamid et al., 2002). In mice, the treatment with estrogen to ovariectomized mice upregulates PCNA expression, while the co-treatment with estrogen and progesterone downregulates PCNA expression (Annie et al., 2019). In addition, we found that the expression levels of metalloproteinase (MMPs) were significantly suppressed in epithelial cells 1 (mouse 2) (Supplementary Table S2). So we preliminarily speculated that the uterus of mouse 2 was in the regenerative stage, and the uterus of mouse1 was in the maturational stage.

To further investigate this speculation, functional enriched terms of the differentially expressed genes in each epithelial cell cluster were shown (Figures 2B,C, Supplementary Table S3). Terms including “response to steroid hormone,” “skin development,” “cellular response to epidermal growth factor stimulus,” “placenta development,” and “response to wounding” were enriched in epithelial cells 2 (mouse 1). These terms showed the active rebuilt of the uterus epithelium to prepare for subsequent fertilization in the maturational status. In this period, we also found terms that indicated “intrinsic apoptotic signaling pathway,” “increased cell mobility,” and “suppressed cell adhesion,” which were all consistent with the changes that happened in the maturational status as previously reported (Yip et al., 2013).

The epithelial cells from two uteri were then clustered exclusively to further verify our hypothesis. We gained 3 clusters (Figure 2D, Supplementary Table S4). According to their *Pcna* expression levels, cluster 1 and cluster 2 showed proliferating characteristics (Figure 2E). We concluded that mouse 2 was in the regenerative stage, for it was entirely composed of proliferating epithelial cells. As for mouse 1, only part of the epithelial cells showed proliferating characteristics (Figure 2F). In addition, the expression level of *Esr1* was suppressed in the epithelial cells of cluster 0 in the mouse 1 (Supplementary Table S4).

Endometrial stromal cells (ESCs) perform a multitude of functions and undertake different functions in different estrous stages (Cottrell et al., 2017). We further explored the characteristics of stromal cells of two mice to further confirm the stages of two mice. The expression levels of MMPs were significantly upregulated in epithelial cells 2 (mouse 1) (Supplementary Table S2), which possibly linked with the active matrix degradation in the maturational stage.

We performed functional enrichment analyses of the differentially expressed genes in each stromal cell cluster to see their functional traits. The term “artery morphogenesis” was enriched in stromal cells in mouse 2 (Figure 2G, Supplementary Table S3). One of the key features of the regeneration stage is angiogenesis. Besides, “protein maturation” and “collagen catabolic process” were activated in this period.

The stromal cells in mouse 1 presented more dynamic cell communications (Figure 2H, Supplementary Table S3). The term “response to progesterone and other steroid hormones” further indicated that mouse 1 was in the maturational stage. The term “response to transforming growth factor beta” was enriched too. Also in the maturational stage, the accelerated cell movement was again seen in this stage, as enrichment analysis suggested the term “positive regulation of cell migration” was significantly enriched. We believed the elevated levels of cell communications and material transportation found in mouse 1 constituted a prosperous metabolism network in stromal cells.

In order to validate the data analysis results, vagina smear and hematoxylin and eosin (H&E) staining were used to determine the mouse estrous cycle (Figure 2I, Supplementary Figure S1) (Bertolin and Murphy, 2014). The expression levels of PCNA in the regenerative mice regenerative endometrium and maturational endometrium were presented (Figure 2I). We found that, like humans, expression level of PCNA in the regenerative endometrium was significantly higher than in the maturational endometrium, indicating the practicability of using PCNA expression level to define the estrus stage.

Taking into account the sheer differences in proliferation and apoptosis, cell adhesion and movement, angiogenesis, and extracellular matrix remodeling, we concluded that mouse 1 was in the maturational stage and mouse 2 was in the regeneration stage.

### The Molecular Trajectory of Stromal Cells in the Two Estrus Stages

ESCs have been reported to perform a multitude of functions including hormonal regulation, decidualization, maternal-fetal communications, and embryo receptivity (Cottrell et al., 2017). After we clarified the estrous stages of two mice, we further explored the characteristics of stromal cells in each estrus period given their significances in the uterus.

**FIGURE 3:**
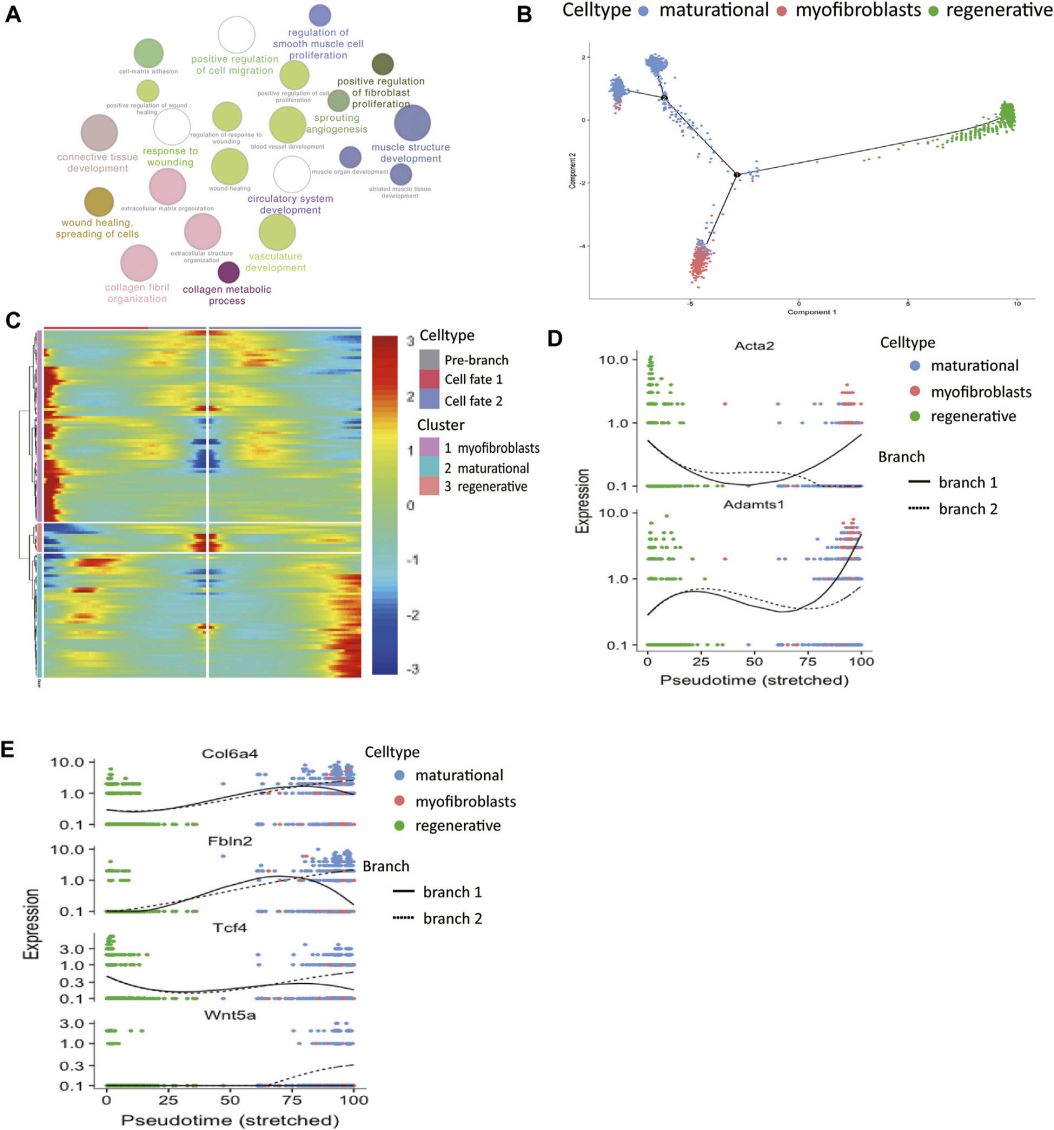
The molecular trajectory of stromal cells in the two estrus stages. **(A)** Gene ontology analysis of differentially upregulated genes in myofibroblasts. **(B)** Monocle generated the pseudotemporal trajectory of stromal cells in different physiological stages. **(C)** Heat map for clustering the significantly branch-1 dependent genes that affected cell fate decisions into three clusters. q < 1E-06. The expression levels of representative genes of **(D)** myofibroblasts and **(E)** maturational stromal cells were shown in the line plots.

Firstly, functional enrichment analysis of upregulated genes of myofibroblasts was performed (Figure 3A, Supplementary Table S3). Terms like “muscle structure development” and “regulation of smooth muscle cell proliferation” confirmed our previous classification of its identity.

The analyses of two stromal subsets in the last section showed that stromal cells in different physiological stages had huge differences in their functions. Therefore, we wanted to further explore how these two cell groups gained their unique traits by differentially gene expression. The pseudotime trajectory of stromal cells in two physiological stages was constructed (Figure 3B). The results showed that there were two differentiation branches: the regenerative stromal cells would transform into myofibroblasts or maturational stromal cells at the first decisional point, and a small part of the maturational cells still had the ability to differentiate into myofibroblasts at the second decisional point. In this way, an estrus cycle was completed. All the significantly expressed branch 1-dependent genes were clustered into three categories by unsupervised clustering (Figure 3C). Among these genes, the myofibroblasts-branch genes had high expression levels of *Acta2* and *Adamts1* (Figure 3D). In the meantime, the maturational_stromal-branch genes had high expression levels of *Col6a4*, *Fbln2*, *Tcf4*, and *Wnt5a* (Figure 3E). These divergent expression patterns of representative genes further confirmed our previous classification. We listed other significant branch-dependent genes, which may also play key roles in the stromal cell differentiation and need further validation (Table 1).

**TABLE 1:** Significant branch-dependent genes of mice stromal cells in different physiological stages.

Regenerative stromal cells	Maturational stromal cells	Myofibroblast
mt-Cytb	Aldh1a2	Adh1
Igfbp7	Hsd11b2	Col3a1
mt-Nd4	Ccl11	Mgp
Igfbp4	Ramp3	Lum
Hba-a1	P2ry14	Cxcl1
Mfap4	Fbln2	Col1a2
mt-Nd2	Crispld2	Fgl2
Id3	Htra1	Has1
Rpl18a	Ramp2	Sparcl1
Itm2b	Jun	Adamts1
Hbb-bs	Atp8a1	Ccl2
	A2m	Mt1
	Dio2	Hk2
	Cd164	Sparc
	Srgn	Acta2
	Sdc1	Il6
	Emb	Col14a1
	Aqp1	Clec3b
	Col6a4	Vcam1
	Mfap5	Pcolce
	Mettl7a1	Ifi205
	Sptssa	Efemp1
	Tcf4	Ly6c1
	Smoc2	Rbp1
	Laptm4a	Gpx3
	Wnt5a	Sqstm1
	Rhob	Dpt
	Hspa1a	Gsn
	Fos	Cxcl16
	Scube1	

### The Immune Landscape of the Uterus During the Two Estrus Stages

**FIGURE 4:**
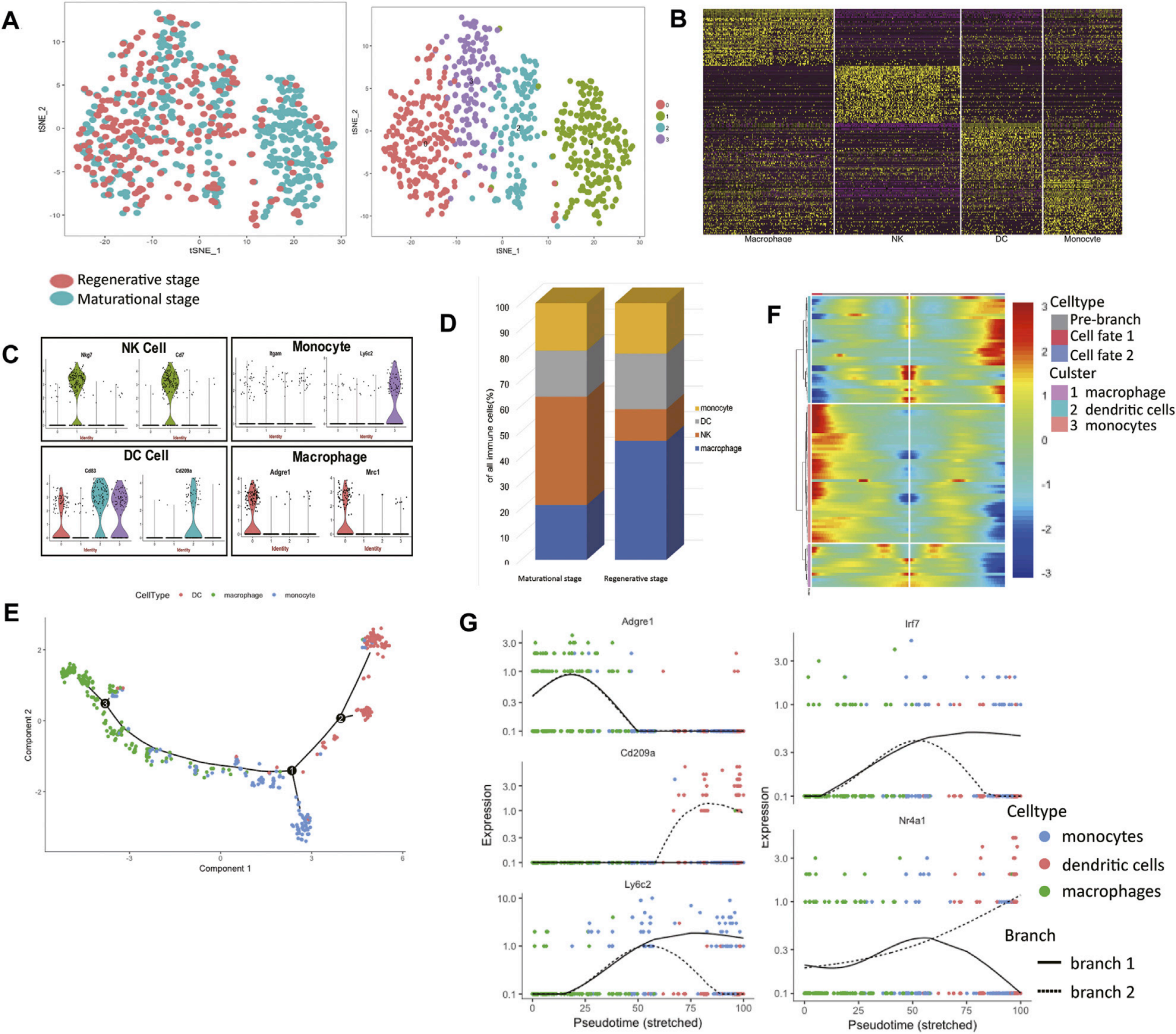
The immune landscape of the uterus during the two estrus stages. **(A)** t-distributed stochastic neighbor embedding (t-SNE) diagram of immune cells in two mice uteri. **(B)** Heatmap of immune cells from the two uteri revealed four populations. **(C)** Violin plots indicating the expression of marker genes of each cell cluster. **(D)** The immune cell composition of the two uteri was different, macrophages dominated in the regenerative stage, while natural killer (NK) cells dominated in the maturational stage. **(E)** Monocle generated the pseudotemporal trajectory of the phagocyte system. **(F)** Heat map for clustering the significantly branch-1 dependent genes that affected cell fate decisions into three clusters. q < 1E-06. **(G)** The expression levels of representative genes of macrophages/dendritic cells (DCs)/monocytes and three transcription factors were shown in the line plots. t-SNE: t-distributed stochastic neighbor embedding; NK: natural killer; DC: dendritic cells.

Embryo implantation and tumor progression are similar to some extent (Holtan et al., 2009). The maternal immune system needs to find the balance and provides an appropriate environment for the fetus to grow. The current findings of NK, monocytes, and dendritic cells (DCs) during the menstrual cycle are limited. For example, findings concerning NK cell number and cytotoxic activity are conflicting (Oertelt-Prigione, 2012). In order to gain reliable results with regard to uterine immune cells at the single-cell level, all immune cells were clustered exclusively into 4 clusters (Figure 4A, Supplementary Table S5). The heatmap of the top 50 markers for each cluster showed that we gained a convincing clustering result (Figure 4B).

Using known markers, the identities of each cluster were clarified (Figure 4C). Cluster 0 expressed *Adgre1* and *Mrc1*, so we clarified them into macrophages. Cluster 1 expressed *Nkg7* and *Cd7*, which made us clarify them into NK cells. Cluster 2 expressed *Cd83* and *Cd209a*, so cells in cluster 2 belonged to DC cells. For their high expression levels of *Ly6c2*, we clarified cluster 3 into monocytes. We found a clear difference in immune cell composition in these two estrus stages. The proportions of macrophages and NK cells in the two stages varied considerably (Figure 4D). Macrophages dominated in the regenerative stage, while NK cells dominated in the maturational stage.

The mechanism of monocytes differentiating into DCs or macrophages is poorly understood. The developmental trajectory of the mononuclear phagocyte system was constructed (Figure 4E). The branch point led to two differentiation paths: macrophages or DCs. Branch-dependent genes were clustered into three categories according to their expression patterns (Figure 4F). The expression levels of marker genes of each branch over pseudotime verified the identities of two differentiation paths (Figure 4G). We listed other significant branch-dependent genes (Table 2). Among them, we detected three transcription factors: *Mafb*, *Irf7*, and *Nr4a1*. *Mafb* was detected in the macrophage branch, *Irf7* expression was seen in the monocyte branch, and *Nr4a1* expressed highly in the DC branch (Figure 4G).

**TABLE 2:** Significant branch-dependent genes of the mononuclear phagocyte system in two mice uteri.

Monocytes	Macrophages	Dendritic cells
Ninj1	Snx2	Cd151
Pilra	C5ar1	Rpl18
Rnh1	Cd83-ps	H2-Eb1
Gm6977	Lgmn	H2-Aa
Card19	Ccl2	Cd74
Ccr1	Csf1r	H2-Ab1
Clec4e	Lst1	Retnlg
Chil3	Mafb	Cxcl16
Thbs1	Lyz2	Rps27
Cd44	Sat1	Rpl18a
Ly6c2	Fcgr3	Cst3
Cd300lf	Ctsb	Rps11
Spp1	C1qb	Rpl32
Mmp19	Apoe	Rps9
Ifitm3	Ctsd	Rps7
Ms4a4c		Rgs1
Lgals3		Nr4a1
Cdkn1a		Bcl2a1d
H3f3b		Cd209a
F10		Tnlp3
Psap		Ccr7
Pkm		Il1b
Lilrb4a		Lsp1
Mdm2		Napsa
Cd14		Mt-Rnr2
Emilin2		Mt-Rnr1
Retnla		Hba-a2
Msr1		Hba-a1
Ms4a6d		Hbb-bs
Clec4d		Ccl4
Ccl9		Hspa1a
Lilr4b		Bcl2a1b
Igkc		Rps21
Hmox1		Rps26
Plin2		Tmsb4x
Fcgr1		Mgp
Cxcl2		Rps29
Ftl1		
Ifi27l2a		
*F*th1		
Irf7		
Ms4a6b		
Cstb		
Fabp5		
Msrb1		
Osm		
Slc11a1		

As for NK cells (Table 3), NK cells in the regenerative stage showed intense inflammatory responses with high expression levels of *Tnf*, *Il1b*, and *S100a8*. It also expressed *Cxcl2* and *Ccrl2*, which respectively promoted the recruitment of neutrophils and themselves (Regan-Komito et al., 2017). In contrast, upregulated genes of NK cells in the maturational stage mostly were immune-suppressive genes like *Serpinb9*, *Stat3*, *Cd96*, and *Cd55*. They also secreted substances that were advantageous for follow-up pregnancy like *Ccl2*.

**TABLE 3:** Significantly differentially expressed genes of immune cells in different physiological stages.

	Upregulated in the regenerative stage	Upregulated in the maturational stage
NK	Tnf	Serpinb9
	Il1b	Stat3
	S100a8	Cd96
	Cxcl2	Cd55
	Ccrl2	Rps6
	Cd74	Gzmb
	H2-Ab1	Lck
	H2-Eb1	Igha
	H2-Aa	Ptpn22
	Ifitm3	Stat3
	Ccl2	Tnfaip3
	Klrg1	Trbc1
	Ly6c1	Ptprc
	Rarres2	Ccl7
	Tgfb1	Tnfrsf1b
Macrophage	Resistin	Il10
	Msrb1	Cd206
	Hes1	Ccl7
	Il1b	Ccl2
	Il18bp	Mrc1
	Mpeg1	Rps6
	S100a8	Cd36
	Msrb1	
	Sox4	
	Retnlg	
	Ccl11	
DC	Ccl22	PD-L1
	Ccl5	Jchain
	Ccrl2	Anxa1
	Itga4	Tnfrsf1b
	Mmp14	Igha
	Irf8	Ccr7
	C1qa	Igkc
	Ccl3	
	Ier3	
Monocyte	S100a8	Thbs1
	Tnf	Ccl2
	Il1b	Ccl7
	Cxcl16	Ccl8
	Ccl3	Lyn
	Mmp14	Anxa1
	Trem2	Cd86
	Mif	Lgals8
	C1qc	Igkc
	H2-K1	Ccl7
	Ccl3	Cd86
	Il1rn	Thbs1
	Hes1	Spp1
	Ccrl2	Cd14
	C1qa	Mif
	Trib1	Jchain
	Cxcl16	

The number of macrophages was higher in the regenerative stage. In this stage, macrophages were pro-inflammatory by secreting resistin, which was a systemic pro-inflammatory cytokine targeting both leukocytes and adipocytes (Table 3) (Nagaev et al., 2006). However, it also showed high expression levels of selenoprotein *Msrb1* and *Hes1* to inhibit inflammatory responses (Lee et al., 2017; Zhang et al., 2019). As for macrophages in the maturational stage, it mainly showed the M2 phenotype with high expression levels of *Il10*, *Cd206*, and *Ccl7*, which were consisted of the characteristics of decidual macrophages (Xuan et al., 2015).

In the regenerative stage, the functions of DC were mainly embodied in influencing other cells. It secreted *Ccl22* to recruit regulatory T (Treg) cells and *Ccl5* to recruit *CCR5*-positive cytokine-induced killer cells (Lee et al., 2016; Yashiro et al., 2019). Its pro-inflammatory role was also embodied in the high expression of *Ccrl2*, which played a significant role in inflammation (Regan-Komito et al., 2017). DCs in the maturational stage were immune-suppressive (Table 3). DCs expressed *PD-L1*, *Jchain*, and *Anxa1*, which were correlated with immune tolerance and refrained the secretion of pro-inflammatory cytokines (Källberg and Leanderson, 2008; Sena et al., 2016).

Monocytes in the maturational stage secreted multiple chemokines like *Thbs1*, *Ccl2*, *Ccl7*, *Ccl8*, *Lyn*, and *Anxa1* to facilitate the migration of themselves (Table 3) (Asano et al., 2015; Liu et al., 2015). Monocytes also expressed M1 markers like *Cd86* and *Lgals8* to enhance inflammatory responses (Patel et al., 2017). By contrast, monocytes in the regenerative stage showed more intense cytolytic ability by producing *S100a8*, *Tnf*, and *Il1b*. It also secreted *Cxcl16* and *Ccl3* to facilitate the recruitment of monocytes and *Mmp14*, *Trem2*, and *Mif* to induce inflammatory responses (Buck et al., 2013; Ruiz-Rosado et al., 2016).

Taken together, we found that the immune responses were suppressed in the maturational stage compared to the regenerative stage by reducing inflammatory cytokines secretion and facilitating differentiation into immune-suppressive cells.

### Identification of the Molecular Feature of Endometrial Mesenchymal Stem Cells *In Vivo*

**FIGURE 5:**
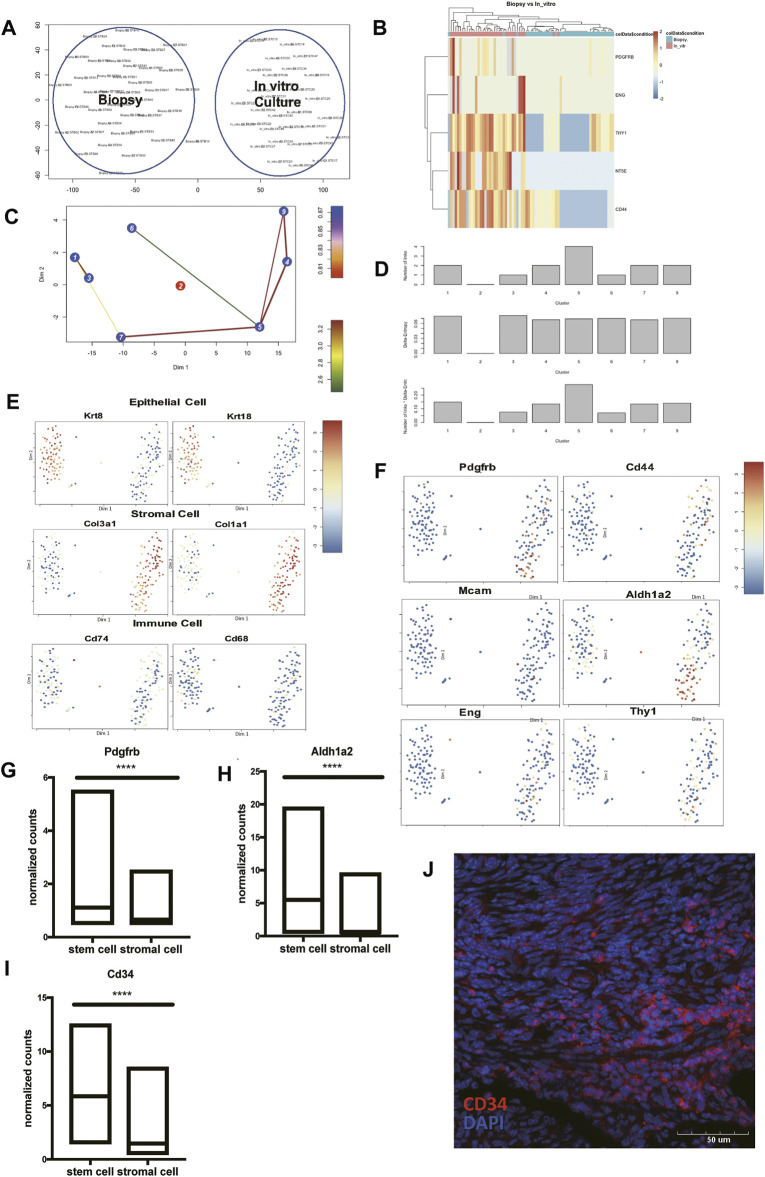
Identify *Pdgfrb* ^*+*^ *Aldh1a2* ^*+*^ *Cd34* ^*+*^ endometrial mesenchymal stem cells *in vivo*. **(A)** Cells directly harvested from biopsy clustered separately with cells after *in-vitro* culture. **(B)** The heat map presented the differentially expressed *Cd73* (*Nt5e*), *Cd90* (*Thy1*), *Cd44*, *Pdgfrb*, and *Eng* in cells directly harvested from biopsy and after *in-vitro* culture. **(C)** Lineage tree generated by StemID2. **(D)** Histograms for the number of links, the delta-entropy, and the StemID2 score generated by StemID2. A high score indicates a higher likelihood that the cluster is an actual stem cell cluster. The t-SNE map with color code representation of log-transformed expression across marker genes of **(E)** epithelial cells, stromal cells, immune cells, and **(F)** stem cells. **(G–I)** The expression level of Pdgfrb, Aldh1a2, and Cd34 in cluster 5 and the rest of the uterine stromal cells. **(J)** Representative IF staining of CD34 expression levels in mice endometrium. (DAPI, blue; CD34, red). Scale bars, 50 μm. IF: immunofluorescence; *p*-value<0.0001.

The mesenchymal-to-epithelial transition has been proposed as a possible reason to explain the periodically endometrial epithelial tissue regeneration (Huang et al., 2012). A previous study has reported a group of cells expressed both the epithelial cell marker, pan-cytokeratin, and the stromal cell marker, vimentin as well (Patterson et al., 2013). We tried to identify multipotent ESCs in the scRNA-seq data using published pipelines (Grün et al., 2016). We failed to find the cell groups that expressed all the proposed stem cell surface molecules like *Cd73*, *Cd90*, *and Cd105*. We hypothesized that expressions of *Cd73*, *Cd90*, and *Cd105* may be induced during *in-vitro* culture. Another scRNA-seq dataset containing cultured ESCs and uncultured ones was used to show the diversity of two culture environments (Krjutškov et al., 2016). The cells from two different culture environments clustered separately (Figure 5A). The stem cell surface markers mentioned above were expressed differently in the two cell groups. We found that stem cell makers like *Cd90* (*Thy1*) and *Cd44* had significant higher expressions in the cultured group (Figure 5B). We found that cell group had a higher entropy score and more connections with other cell types (Figures 5C,D,F, Supplementary Table S6), had the potential to differentiate into stromal cells, epithelial cells, and immune cells (Figure 5E), which make it the potential multipotent ESCs *in vivo*. This cell subcluster expressed higher expression of *Cd34*, *Pdgfrb*, and *Aldh1a2* (Figures 5F–I). The representative immunofluorescence staining of CD34 in mice endometrium was shown, indicating the existence of this endometrial mesenchymal stem cells (Figure 5J).

To conclude, we found the transcriptional signature of endometrial mesenchymal stem cells *in vivo*. And the cell group 5 that we presented here showed a great possibility to be the cells responsible for re-epithelialization.

## Discussion

There is still a huge void in uterus-related research at the single-cell level. In humans, the sequence data of *Cd13* ^+^ stromal and *Cd9* ^*+*^ epithelial cells from endometrial tissues have been generated (Krjutškov et al., 2016). Researchers also used scRNA-seq to map the temporal transcriptomic changes in cultured primary ESCs along a decidual time-course and in response to the withdrawal of differentiation signals (Lucas et al., 2020). In mice, the transcriptional profiles of uterine epithelial cells at five developmental stages, ranging from neonatal to mature stages were analyzed (Wu et al., 2017). These studies contained a limited number of cell types. A scMCA covering major cell types was completed (Han et al., 2018). It included the uterus and mice other major organs as well, yet without an in-depth analysis of the changes that happened in the endometrium at different estrus stages and left opportunities for later researchers to delineate the dynamic endometrial cell transformation of all cell types in the estrus cycle at the single-cell level. Recently, mesenchymal cells in the adult mouse endometrium have been characterized and five subpopulations have been identified (Kirkwood et al., 2021). Our research is a systematical single-cell level study with a special focus on constructing a mice cell atlas at different estrus stages and showed that different estrus stages had sheer different cell composition.

We identified three transcription factors in the differentiation path of the mononuclear phagocyte system*. Mafb* was reported to be essential for monocyte-macrophage differentiation in the previous study (Goudot et al., 2017). *C1qb*, as the complement component of C1q complex, was one of the *Mafb* target genes and conformably upregulated in the macrophage branch (Table 2) (Hamada et al., 2020).

*Irf7* has been reported to be involved in the regulatory pathway initiated in DCs during their response to microbial stimuli but dispensable in DC development (Owens et al., 2012). *Nr4a1* has been reported to be the target for modulating the inflammatory phenotypes of monocytes and macrophages (Hanna et al., 2012). In our analyses, *Irf7* expression was seen in the monocyte branch, and *Nr4a1* expressed highly in the DC branch, so the relations between these two transcription factors and their branches needed further experiments. Likewise, understanding the decisional mechanism of fibroblast-to-myofibroblast differentiation may be the key to tackle endometriosis and adenomyosis. Among the highly expressed genes that determined the myofibroblast differentiation (Table 1), there were a lot of them have not been reported, which shed light on future research.

Generally, the immune cells in the uterus endometrium showed a pro-inflammatory tendency in the regenerative stage and an anti-inflammatory tendency in the maturational stage. Yet the macrophages in the regenerative stage also expressed certain immune-suppressive genes, indicating the possibility that tissue-resident macrophages existed and contributed to endometrium repair. The findings concerning uterus tissue-resident macrophages are rare. Putative tissue-resident macrophages have been reported to be spatially restricted and in association with areas of repaired, re-epithelialized endometrium (Cousins et al., 2016). In our pseudotime analysis (Figure 4E), we found a branch in the path of macrophage differentiation, which indicated the existence of a subtype of macrophages and might be the uterus tissue-resident macrophages. The molecular profiles of mouse uterus tissue-resident macrophages remained unknown, which needed further research. Our results may provide insights into mechanisms regarding tissue remodeling and aid in tackling endometrium abnormalities like endometritis.

We reported a novel group of markers for *in vivo* endometrial mesenchymal stem cells: *Pdgfrb*, *Aldh1a2*, and *Cd34*, which has not been reported before. *Cd34* ^*+*^ *Klf4* ^*+*^ stromal-resident stem cells have been reported to directly contribute to endometrial regeneration (Yin et al., 2019). *PDGFRα* ^+^/*CD34* ^+^ Celll group 5 highly expressed *Cd34*, but without detectable expression of *Klf4*. Many proposed stem cell surface molecules like *Cd73*, *Cd90*, and *Cd105* failed to express highly in *in vivo* endometrial mesenchymal stem cells (Kyurkchiev et al., 2010).

However, several limitations of our study should be acknowledged. Firstly, this is an RNA-seq based bioinformatics study and caution must be taken when further extrapolating these results *in vivo.* Future study is needed to evaluate the presence of a transcript corresponds to its expression at the protein level. Experiments such as further functional verification of reported endometrial mesenchymal stem cells are crucial in the future. Secondly, the limited number of mice that participated in this study may affect the statistical power and the strength of our conclusions. Thirdly, one of our goals in this study is to seek insights from mice scRNA-seq data and shed light on human endometrium research. However, species-specificities in uterine physiology exist, which may dampen the practicability of our study.

**FIGURE 6:**
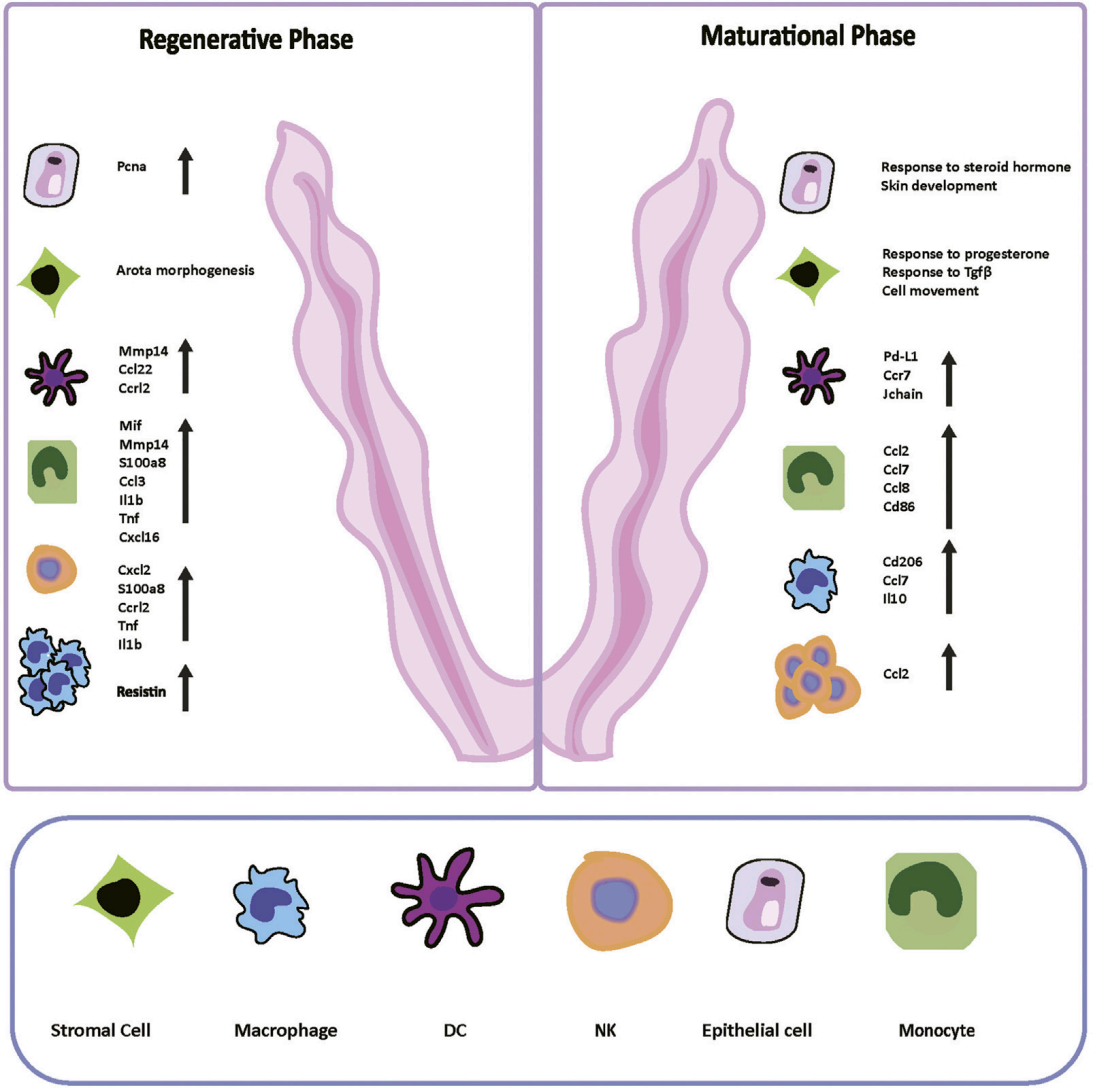
Digital Cell Atlas of Mouse Uterus: from regenerative stage to maturational stage. Based on the *Pcna* expression, differentially expressed genes, and gene ontology terms of two stages, we built up the cell atlas of the mouse uterus from the regenerative to maturational endometrium. The number and function of immune cells varied sharply in two periods. In the regenerative stage, mice epithelial cells expressed Pcna highly, and the term “aorta morphogenesis” was enriched in stromal cells. Macrophages dominated in this stage. The immune system was boosted with intensely secreted cytokines and chemokines. In the maturational stage, cells presented more dynamic cell communication instead of proliferating. Terms “response to progesterone and other steroid hormones” and “response to transforming growth factor beta” were enriched. NK cells dominated in this stage. The immune responses were suppressed compared to the regenerative stage by reducing inflammatory cytokines secretion and facilitating differentiation into immune-suppressive cells. *Pcna*: proliferating cell nuclear antigen; NK: natural killer; DC: dendritic cells.

We thoroughly compared the functional traits and molecular profiles of each cell type from the regenerative stage to the maturational stage (Figure 6). With a template for the interactions of different cell types at the normal condition, researchers can better identify morphologically unaffected abnormalities in future studies. We also depicted the transitions and transcription factors that shaped the differentiation of ESCs and the mononuclear phagocyte system, and many of them have not been reported before. The genes that have not been reported before can inspire subsequent basic research. The cell atlas of mice uteri presented here would improve our understanding of the functional changes that occurred in the endometrium during the estrus cycle.

## Conclusion

This study is a systematical single-cell level study to construct a mice cell atlas from the regenerative stage to the maturational stage, including epithelial cells, stromal cells, and immune cells. The functional traits and molecular profiles for each cell type in these two stages are thoroughly compared. The mice cell atlas also delineates the transitions that shape the differentiation of endometrial stromal cells and the mononuclear phagocyte system. This study found putative uterus tissue-resident macrophages and *Pdgfrb* ^*+*^ *Aldh1a2* ^*+*^ *Cd34* ^*+*^ endometrial mesenchymal stem cells *in vivo*.

## Data Availability

The original contributions presented in the study are included in the article/Supplementary Material, further inquiries can be directed to the corresponding author.

## Supplementary Material

Supplementary Figure S1
**(A)** Scheme of collecting vaginal smears in 6 consecutive days before and at harvest day. Representative images of vaginal smears of **(B)** regenerative endometrium and **(C)** maturational endometrium. Scale bars, 200 μm.

## Abbreviations

ESC, Endometrial stromal cells; PCA, principal component analysis; t-SNE, t-distributed stochastic neighbor embedding; GO, Gene Ontology; MMP, Metalloproteinase; NK, natural killer; IFN-γ, interferon-γ; DCs, dendritic cells; Treg, regulatory T cells; scRNA-seq, single-cell RNA sequencing; Pcna, proliferating cell nuclear antigen.
