# Supplementary figures and images for: Digital Cell Atlas of Mouse Uterus: From Regenerative Stage to Maturational Stage
Source: Front Genet. 2022 May 20;13:847646. doi: 10.3389/fgene.2022.847646 (PMC9163836; doi:10.3389/fgene.2022.847646)

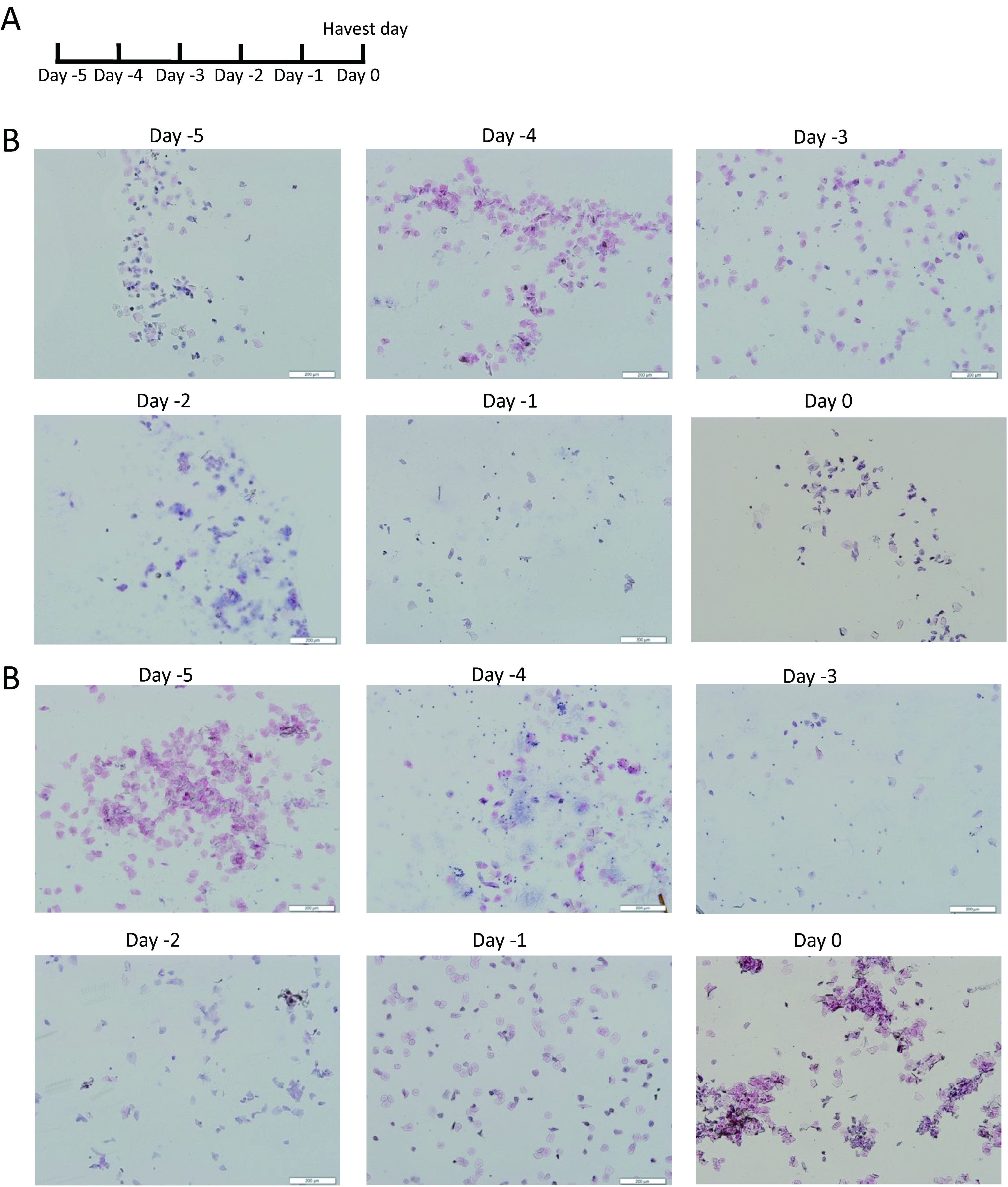

Supplement: Supplementary file 3 [file Image1.TIF]
